# Global, regional, and national burden of pulmonary arterial hypertension from 1990 to 2021 and projection to 2050: A systematic analysis for the global burden of disease study 2021

**DOI:** 10.1371/journal.pone.0338335

**Published:** 2025-12-29

**Authors:** Changyong Wu, Zhongkai Wang, Yihua Luo, Huang Sun, Ruijie Li, Weijie Liu, Fei Xu, Bingqing Zhang, Yunzhu Peng

**Affiliations:** 1 Department of Cardiology, The First Affiliated Hospital of Kunming Medical University, Kunming, China; 2 Department of Emergency, The First Affiliated Hospital of Kunming Medical University, Kunming, China; 3 Department of Cardiology, The Affiliated Hospital of Yunnan University, Kunming, China; Gent University, BELGIUM

## Abstract

**Background:**

Pulmonary arterial hypertension (PAH) is a progressive and incurable syndrome characterized by pulmonary vascular remodeling. Although targeted therapies have advanced, prognosis remains poor, underscoring the need for comprehensive epidemiological evaluations to guide public health strategies and resource allocation.

**Methods:**

This study assessed the global, regional, and national burden of PAH from 1990 to 2021 using data from the Global Burden of Disease Study (GBD) 2021. Metrics included prevalence, incidence, mortality, and disability-adjusted life years (DALYs), with stratified assessments by geography, gender, age, and socio-demographic index (SDI).

**Results:**

In 2021, the global PAH burden comprised 191,808 prevalent cases, 43,251 incident cases, 22,021 deaths, and 642,104 DALYs. Age-standardized rates declined consistently over the 32-year period. Population growth was the prominent contributor of the PAH burden, with higher rates in females and older age groups. Prevalence, mortality, and DALYs decreased with higher SDI, whereas incidence showed an inverse trend. The disparity between high and low SDI countries widened, with the slope index of inequality increasing from −5.21 in 1990 to −1.52 in 2021. Predictions revealed that further declines in age-standardized rates of prevalence, mortality, and DALYs from 2022 to 2025, but a rise in incidence.

**Conclusions:**

A decline in the age-standardized rates of PAH was observed, whereas a persisting high absolute disease burden was evident, clustering among women, older populations, and low-SDI regions.

## 1. Introduction

Pulmonary arterial hypertension (PAH) is characterized by elevated mean pulmonary arterial pressure (mPAP) ≥20 mmHg [1]. The World Health Organization (WHO)  classifies PAH into 5 distinct groups [2]. Recent data indicated that the average hospitalization charge for PAH patients rose significantly from $82,000 in 2016 to $125,000 in 2020 [3]. Analyses of commercial insurance databases further revealed that marked differences in monthly treatment expenditures, with mean expenditures of $9,503 for monotherapy and $16,240 for combination therapy [3]. These indicators highlighted the progressive evolution of PAH into formidable global public health challenge.

Although advances in targeted molecular therapies have improved clinical outcomes, persistent disparities in disease burden across demographic and socioeconomic groups remained a concern [4,5]. Present estimates for PAH in developed countries indicated an annual incidence of 1.1–7.6 per million adults and a prevalence of 6.6–26.0 per million adults [6]. For instance, a cohort of over 60 million patients from the United States healthcare database reported a PAH prevalence of 0.1 [7]. A systematic review published in Global Index Medicus complied data from 6,772 patients from 1980 to 2021, summarizing prevalence, incidence, and 1-year survival, yet which did not addressed mortality and disability-adjusted life years (DALYs) [8]. Consequently, a comprehensive and updated assessment of global burden and temporal trends of PAH remained lacking, which may be partly explained by the evolving definition and threshold of PAH.

The study utilized data from the Global Burden of Disease Study (GBD) 2021 to systemically assess the global epidemiology of PAH. Through a comprehensive analysis across global, regional, and national levels, we focused on PAH burden and trend in prevalence, incidence, mortality, and DALYs from 1990 to 2021.

## 2. Materials and methods

### 2.1. Data source and disease definition

Data were derived from GBD 2021, which provides comprehensive estimates for 288 causes of death, 371 diseases and injuries, as well as risk factors across 21 GBD regions and 204 countries and territories [9]. All original data were sourced from the Global Health Data Exchange (https://vizhub.healthdata.org/gbd-results/). Ethical approval for this study was not required, as the University of Washington Institutional Review Board has waived the requirement for informed consent-access the GBD data [10]. The GBD estimation process employed standardized approach to calculate health metrics, including prevalence, incidence, mortality, and DALYs, reporting both absolute numbers and age-standardized rates per 100,000 populations. Case numbers were directly provided in GBD 2021, with 95% uncertainty interval (UI) derived from the 2.5^th^ and 97.5^th^ percentiles of 1,000 algorithmically generated estimate iterations.

PAH constitutes a complex clinical disorder characterized by progressive structural remodeling and functional impairment of the pulmonary vascular bed, ultimately leading to right heart failure and death. Within the GBD 2021 framework, PAH is classified under WHO group 1 pulmonary hypertension. The diagnostic criteria of PAH were updated in 2018 to define an mPAP > 20mmHg at rest, as measured by right heart catheterization (RHC) [11]. Similarly, the GBD defines PAH cases as those with clinical diagnosis confirmed by evidence from RHC or echocardiography. Based on pulmonary arterial wedge pressure (PAWP) and pulmonary vascular resistance (PVR), PAH is divided into three categories: pre-capillary pulmonary hypertension (mPAP > 20mmHg, PAWP ≤15 mmHg, and PVR ≥ 2 Wood units), isolated post-capillary pulmonary hypertension (mPAP > 20mmHg, PAWP >15 mmHg, and PVR ≤ 2 Wood units), and combined post-and pre-capillary pulmonary hypertension (mPAP > 20mmHg, PAWP >15 mmHg, and PVR > 2 Wood units) [12].

### 2.2. Socio-demographic index (SDI)

SDI quantifies regional development status through integrating three demographic parameters: gross domestic product per capita, population mean educational attainment, and age-specific fertility rates among women aged <25 years [13]. The composite metric demonstrates strong positive correlations with enhanced socioeconomic development indices and superior populations health indicators. In accordance with GBD 2021 datasets, the 204 nations and territories were stratified into quintile SDI categories: low (0–0.454743), low-middle (0.454743–0.607679), middle (0.607679–0.689054), high-middle (0.689054–0.805129), and high (0.805129–1) [9].

### 2.3. Statistical analysis

First, annual percent change (APC) and average annual percent change (AAPC) were calculated by the Joinpoint regression analysis model [13]. From a statistical standpoint, an APC or AAPC estimate, alongside its 95% confidence interval (CI) lower limit exceeding 0, denoted an upward trajectory in the specified interval. In contrast, an APC or AAPC estimate coupled with a 95% CI upper limit falling below 0 signals a downward trend. When the 95% CI for the APC or AAPC encompasses 0, it implied that there was no statistically significant difference in trend shifts. Second, Spearman correlation analysis was applied to assess the association between SDI and age-standardized rates of PAH, and the correlation coefficient that ranged from −1 to 1 described the strength and direction of their linear relationship across various geographies. In addition, we conducted a decomposition analysis to explore the drivers of PAH from 1990 to 2021 [14]. Then, health inequality analysis was employed to measure the distributive inequality of PAH burden across 204 countries [15]. Finally, we utilized the Bayesian age-period-cohort (BAPC) model with integrated nested Laplace approximation to delineate and project PAH trends up to 2050 [16,17]. All analyses and visualizations were performed utilizing R software (Version 4.3.3) and Stata software (Version 18 MP).

## 3. Results

### 3.1. Global level

According to GBD 2021 estimates, there were 191,808 prevalent cases of PAH (95% UI: 155,357−235,787) globally, with an age-standardized prevalence rates (ASPR) of 2.28 per 100,000 populations (95% UI: 1.85–2.80). From 1990 to 2021, the ASPR remained stable, with a minimal change of −0.01 per 100,000 populations (95% UI: −0.02 to 0.00). A total of 43,251 new cases were recorded (95% UI: 34,705−52,441), corresponding to an age-standardized incidence rates (ASIR) of 0.52 per 100,000 populations (95% UI: 0.42–0.62). The ASIR showed a slight increase of 0.03 per 100,000 populations (95% UI: 0.03 to 0.04) over the 32-year period. Mortality documented 22,021 deaths (95% UI: 18,239−25,351), with an age-standardized mortality rates (ASMR) of 0.27 per 100,000 populations (95% UI: 0.23–0.32). The ASMR declined significantly since 1990, with a net change of −0.22 per 100,000 populations (95% UI: −0.35 to −0.08). The disease burden measured in DALYs was 642,104 (95% UI: 552,273−728,993). The age-standardized DALYs rates (ASDR) was 8.24 per 100,000 populations (95% UI: 7.14–9.39), reflecting a substantial reduction of −0.38 (95% UI: −0.48 to −0.25) between 1990 and 2021 (Table 1 and S1 Fig).

**Table 1:** Cases of prevalence, incidence, deaths, and DALYs for PAH in 2021 for both sexes and rate change of age-standardized rates by GBD.

Location	Prevalence (95% UI)			Incidence (95% UI)				Deaths (95% UI)			DALYs (95% UI)		
	Counts (2021)	ASR per 100,000 (2021)	Change in ASR, 1990–2021	Counts (2021)	ASR per 100,000 (2021)		Change in ASR, 1990–2021	Counts (2021)	ASR per 100,000 (2021)	Change in ASR, 1990–2021	Counts (2021)	ASR per 100,000 (2021)	Change in ASR, 1990–2021
Global	191,808 (155,357–235,787)	2.28 (1.85–2.80)	−0.01 (−0.02 to 0.00)	43,251 (34,705–52,441)	0.52 (0.42–0.62)		0.03 (0.03 to 0.04)	22,021 (18,239–25,351)	0.27 (0.23–0.32)	−0.22 (−0.35 to −0.08)	642,104 (552,273–728,993)	8.24 (7.14–9.39)	−0.38 (−0.48 to −0.25)
Low SDI	15,181 (12,474–18,747)	1.94 (1.58–2.36)	−0.06 (−0.08 to −0.05)	5,712 (4,685–6,847)	0.71 (0.58–0.85)		−0.09 (−0.10 to −0.08)	1,782 (1,147–2,544)	0.27 (0.15–0.40)	−0.16 (−0.34 to 0.10)	95,342 (67,471–133,050)	9.30 (6.08–13.20)	−0.25 (−0.43 to −0.07)
Low-Middle SDI	32,715 (26,546–40,620)	1.90 (1.53–2.33)	0.07 (0.06 to 0.09)	9,984 (8,086–12,020)	0.59 (0.47–0.70)		−0.02 (−0.03 to −0.01)	3,728 (2,757–5,091)	0.26 (0.18–0.38)	−0.25 (−0.39 to −0.08)	156,400 (122,426–194,166)	9.07 (7.05–11.60)	−0.39 (−0.51 to −0.21)
Middle SDI	59,667 (48,065–73,648)	2.21 (1.80–2.71)	0.07 (0.06 to 0.08)	14,174 (11,319–17,383)	0.53 (0.43–0.64)		−0.01 (−0.01 to <0.01)	7,548 (5,141–9,026)	0.33 (0.22–0.39)	−0.28 (−0.50 to −0.02)	197,171 (148,781–232,321)	8.23 (6.26–9.70)	−0.42 (−0.58 to −0.25)
High-Middle SDI	42,636 (34,236–52,932)	2.54 (2.07–3.12)	−0.03 (−0.04 to −0.02)	7,734 (6,187–9,586)	0.46 (0.37–0.55)		0.06 (0.05 to 0.07)	4,326 (3,594–5,141)	0.24 (0.20–0.29)	−0.32 (−0.45 to −0.14)	99,448 (85,757–11,763,9)	6.48 (5.61–7.87)	−0.51 (−0.62 to −0.35)
High SDI	41,452 (33,445–51,588)	2.64 (2.15–3.23)	−0.01 (−0.02 to <0.01)	5,612 (4,500–6,959)	0.37 (0.29–0.44)		−0.01 (−0.02 to <0.01)	4,621 (3,919–5,054)	0.22 (0.19–0.23)	−0.17 (−0.25 to −0.08)	93,182 (84,873–99,192)	6.16 (5.76–6.49)	−0.33 (−0.40 to −0.27)
Central Asia	2,162 (1,758–2,677)	2.33 (1.90–2.85)	−0.05 (−0.07 to −0.03)	399 (320–487)	0.44 (0.36–0.54)		0.07 (0.05 to 0.09)	319 (261–382)	0.41 (0.34–0.48)	0.04 (−0.13 to 0.26)	11,619 (9,514–14,202)	12.91 (10.61–15.60)	−0.09 (−0.25 to 0.13)
Central Europe	3,521 (2,864–4,311)	2.30 (1.89–2.79)	−0.09 (−0.10 to −0.07)	794 (644–986)	0.47 (0.38–0.57)		0.20 (0.19 to 0.22)	438 (398–479)	0.21 (0.19–0.23)	−0.18 (−0.28 to −0.04)	10,424 (9,512–11,459)	6.05 (5.5–6.67)	−0.26 (−0.35 to −0.14)
Eastern Europe	7,686 (6,264–9,489)	2.83 (2.32–3.43)	−0.12 (−0.13 to −0.10)	1,198 (962–1,488)	0.43 (0.35–0.52)		0.15 (0.13 to 0.17)	278 (258–300)	0.09 (0.08–0.10)	−0.62 (−0.67 to −0.56)	8,357 (7,760–8,996)	3.32 (3.10–3.56)	−0.64 (−0.69 to −0.59)
Australasia	1,167 (936, 1,444)	2.83 (2.29–3.44)	−0.05 (−0.09 to −0.01)	153 (123–190)	0.37 (0.30–0.45)		0.07 (0.03 to 0.11)	58 (49–65)	0.11 (0.10–0.13)	−0.45 (−0.58 to −0.36)	1,434 (1,305–1,563)	3.67 (3.38–3.99)	−0.50 (−0.61 to −0.42)
High-income Asia Pacific	9,167 (7,427–11,362)	3.01 (2.47–3.68)	−0.07 (−0.09 to −0.06)	942 (761–1,175)	0.33 (0.26–0.40)		0.06 (0.04 to 0.07)	1,049 (826–1,201)	0.23 (0.20–0.26)	−0.10 (−0.19 to −0.02)	19,988 (17,442–21,997)	8.31 (7.74–8.87)	−0.31 (−0.37 to −0.25)
High-income North America	8,625 (6,918–10,745)	1.73 (1.40–2.12)	−0.08 (−0.09 to −0.06)	1,524 (1,209–1,891)	0.30 (0.24–0.37)		0.12 (0.11 to 0.14)	1,880 (1,620–2,043)	0.29 (0.26–0.31)	−0.08 (−0.16 to 0.01)	38,373 (35,060–40,844)	7.71 (7.17–8.18)	−0.24 (−0.31 to −0.18)
Western Europe	23,621 (19,121–29,197)	3.56 (2.92–4.35)	0.11 (0.09 to 0.13)	2,593 (2,092–3,209)	0.41 (0.33–0.49)		−0.10 (−0.12 to −0.09)	1,788 (1,533–1,943)	0.18 (0.16–0.19)	−0.24 (−0.34 to −0.15)	34,043 (31,024–36,440)	5.05 (4.75–5.32)	−0.39 (−0.46 to −0.34)
Caribbean	1,229 (987–1,506)	2.39 (1.93–2.93)	−0.07 (−0.09 to −0.04)	247 (199–299)	0.48 (0.39–0.58)		0.06 (0.04 to 0.08)	95 (66–130)	0.20 (0.13–0.29)	−0.47 (−0.59 to −0.32)	5,071 (2,988–7,877)	11.73 (6.48–18.74)	−0.41 (−0.56 to −0.22)
Central Latin America	8,421 (6,856–10,383)	3.25 (2.64–3.99)	0.20 (0.17 to 0.22)	1,267 (1,024–1,535)	0.49 (0.40–0.59)		−0.13 (−0.14 to −0.11)	201 (177–230)	0.08 (0.07–0.10)	−0.48 (−0.57 to −0.37)	7,246 (6,391–8,407)	3.00 (2.63–3.51)	−0.51 (−0.61 to −0.41)
Andean Latin America	1,777 (1,434–2,194)	2.77 (2.24–3.40)	−0.05 (−0.08 to −0.02)	347 (282–418)	0.55 (0.44–0.66)		0.03 (0.01 to 0.07)	91 (72–119)	0.16 (0.12–0.20)	−0.45 (−0.57 to −0.26)	3,544 (2,795–4,490)	5.73 (4.52–7.28)	−0.52 (−0.65 to −0.26)
Southern Latin America	2,227 (1,800–2,760)	2.82 (2.29–3.48)	0.05 (0.01 to 0.08)	259 (208–320)	0.33 (0.27–0.41)		−0.02 (−0.05 to 0.01)	150 (138–162)	0.18 (0.17–0.20)	−0.49 (−0.56 to −0.41)	4,739 (4,439–5,092)	6.55 (6.13–7.07)	−0.60 (−0.65 to −0.54)
Tropical Latin America	6,239 (5,020–7,679)	2.48 (2.00–3.04)	0.06 (0.04 to 0.07)	1,226 (984–1,492)	0.49 (0.39–0.59)		−0.02 (−0.03 to −0.01)	779 (714–822)	0.32 (0.29–0.34)	−0.14 (−0.2 to −0.08)	24,235 (23,002–25,403)	10.22 (9.65–10.78)	−0.28 (−0.34 to −0.22)
North Africa and Middle East	11,591 (9,389–14,436)	2.03 (1.64–2.49)	<0.01 (−0.02 to 0.02)	2,881 (2,326–3,524)	0.52 (0.42–0.63)		−0.08 (−0.09 to −0.06)	1,896 (1,328–2,305)	0.44 (0.31–0.53)	−0.42 (−0.57 to −0.20)	80,753 (58,086–98,810)	14.81 (10.76–17.96)	−0.59 (−0.73 to −0.40)
South Asia	29,561 (23,835–36,927)	1.71 (1.38–2.09)	0.08 (0.06 to 0.09)	9,520 (7,651–11,461)	0.56 (0.45–0.67)		<0.01 (−0.01 to 0.01)	3,549 (2,321–5,532)	0.25 (0.16–0.42)	−0.19 (−0.38 to 0.04)	136,563 (97,809–189,353)	8.54 (6.02–12.46)	−0.30 (−0.44 to −0.10)
East Asia	42,486 (33,930–53,043)	2.23 (1.81–2.75)	0.08 (0.06 to 0.09)	9,572 (7,599–11,898)	0.50 (0.40–0.60)		−0.02 (−0.03 to −0.01)	7,490 (4,986–9,266)	0.41 (0.28–0.50)	−0.31 (−0.55 to 0.01)	154,740 (102,939–190,399)	8.84 (5.99–11.01)	−0.44 (−0.65 to −0.20)
Southeast Asia	13,616 (11,019–16,957)	1.90 (1.54–2.32)	0.07 (0.06 to 0.09)	4,082 (3,268–4,966)	0.58 (0.47–0.69)		0.05 (0.04 to 0.07)	741 (525–1,850)	0.12 (0.08–0.32)	−0.21 (−0.39 to 0.10)	31,112 (22,913–58,708)	4.65 (3.39–9.25)	−0.26 (−0.43 to <0.01)
Oceania	196 (159–241)	1.80 (1.45–2.17)	−0.09 (−0.13 to −0.06)	65 (53–79)	0.61 (0.49–0.73)		0.08 (0.04 to 0.12)	25 (17–43)	0.24 (0.16–0.48)	−0.14 (−0.31 to 0.06)	1,455 (988–2,411)	10.14 (6.90–17.10)	−0.07 (−0.27 to 0.20)
Central Sub-Saharan Africa	1,693 (1,378–2,094)	1.86 (1.49–2.24)	−0.34 (−0.37 to −0.30)	776 (636–938)	0.83 (0.67–0.98)		0.02 (−0.01 to 0.06)	131 (62–237)	0.19 (0.08–0.37)	−0.21 (−0.4 to 0.05)	6,524 (3,586–11,025)	6.16 (2.94–11.09)	−0.32 (−0.55 to −0.07)
Eastern Sub-Saharan Africa	5,907 (4,822–7,344)	2.09 (1.70–2.53)	−0.13 (−0.15 to −0.12)	2,687 (2,209–3,243)	0.92 (0.75–1.09)		−0.07 (−0.08 to −0.06)	468 (219–878)	0.18 (0.07–0.34)	−0.35 (−0.46 to −0.14)	26,605 (14,265–49,615)	6.85 (3.30–12.62)	−0.38 (−0.53 to −0.19)
Southern Sub-Saharan Africa	1,596 (1,287–1,945)	2.28 (1.85–2.76)	0.08 (0.05 to 0.10)	537 (439–649)	0.75 (0.61–0.90)		−0.08 (−0.1 to −0.06)	72 (53–86)	0.11 (0.08–0.13)	−0.07 (−0.29 to 0.21)	3,212 (2,359–3,891)	4.33 (3.21–5.20)	−0.08 (−0.28 to 0.14)
Western Sub-Saharan Africa	9,319 (7,635–11,570)	2.64 (2.15–3.23)	0.23 (0.19 to 0.27)	2,181 (1,786–2,634)		0.64 (0.52–0.78)	−0.23 (−0.24 to −0.21)	523 (306–774)	0.17 (0.07–0.28)	−0.33 (−0.52 to −0.07)	32,071 (22,084–47,222)	6.50 (3.77–9.47)	−0.30 (−0.48 to −0.08)

ASR: age-standardized rates, UI: uncertainty interval.

### 3.2. Regional level

The GBD 2021 recorded that high SDI regions exhibited the highest ASPR at 2.64 per 100,000 populations (95% UI: 2.15–3.23), while low-middle SDI regions had the lowest ASPR at 1.90 per 100,000 populations (95% UI: 1.53–2.33). Geographically, Western Europe recorded the highest ASPR (3.56, 95% UI: 2.92–4.35), followed by Central Latin America (3.25, 95% UI: 2.64–3.99). In contrast, South Asia had the lowest ASPR (1.71, 95% UI: 1.38–2.09), marginally below High-income North America (1.73, 95% UI: 1.40–2.12). Between 1990 and 2021, ASPR revealed divergent trajectories: low-middle and middle SDI regions experienced a slight increase at 0.07 per 100,000 populations. Western Sub-Saharan Africa (0.23, 95% UI: 0.19 to 0.27) and Central Latin America (0.20, 95% UI: 0.17 to 0.22) showed significant upward trends. The most notable decline occurred in Central Sub-Saharan Africa (−0.34, 95% UI: −0.37 to −0.30), followed Eastern Sub-Saharan Africa (−0.13, 95% UI: −0.15 to −0.12) and Eastern Europe (−0.12, 95% UI: −0.13 to −0.10). Moreover, the highest ASIR was in Eastern Sub-Saharan Africa (0.92, 95% UI: 0.75–1.09) and Central Sub-Saharan Africa (0.83, 95% UI: 0.67–0.98) (Table 1 and S1 Fig).

The ASMR also showed regional variation. Middle SDI regions experienced the greatest increase (0.33, 95% UI: 0.22–0.39), whereas high-middle SDI regions saw the largest decline (−0.32, 95% UI: −0.45 to −0.14). Geographically, the highest ASMR values was reported in North Africa and Middle East (0.44, 95% UI: 0.31–0.53), East Asia (0.41, 95% UI: 0.28–0.50), and Central Asia (0.41, 95% UI: 0.34–0.48), respectively. It was noteworthy that a slight increasing trend of ASMR from 1990 to 2021 was in Central Asia (0.04, 95% UI: −0.13 to 0.26), while Eastern Europe showed the most pronounced decrease (−0.62, 95% UI: −0.67 to −0.56) (Table 1 and S1 Fig).

Globally, the ASDR declined from 1990 to 2021. In 2021, the highest ASDR was observed in low SDI regions (9.30, 95% UI: 6.08–13.20), and the lowest in high SDI regions (6.16, 95% UI: 5.76–6.49). Significant reduction occurred in Eastern Europe (−0.64, 95% UI: −0.69 to −0.59), Southern Latin America (−0.60, 95% UI: −0.65 to −0.54), and North Africa and Middle East (−0.59, 95% UI: −0.73 to −0.40). Conversely, the highest regional ASDR values were reported in North Africa and Middle East (14.81, 95% UI: 10.76–17.96), Central Asia (12.91, 95% UI: 10.61–15.60), and Caribbean (11.73, 95% UI: 6.48–18.74) (Table 1 and S1 Fig).

Correlation analysis elucidated a moderately strong positive relationship between SDI and ASPR (R = 0.51, *P* < 0.001), and a negative correlation with ASIR (R = −0.84, *P* < 0.001). Associations between SDI and ASMR and ASDR were also observed but were less pronounced than for ASIR (S2 Fig).

### 3.3. National level

In 2021, Switzerland (7.09, 95% UI: 5.80–8.66), Sweden (6.30, 95% UI: 5.19–7.70), and Netherlands (4.66, 95% UI: 3.79–5.76) recorded the highest ASPR of PAH. Conversely, Greenland (1.32, 95% UI: 1.06–1.62), Pakistan (1.38, 95% UI: 1.11–1.70), and Afghanistan (1.46, 95% UI: 1.18–1.78) exhibited the lowest ASPR. From 1990 to 2021, Nigeria (0.53, 95% UI: 0.48–0.60), El Salvador (0.47, 95% UI: 0.36–0.57), and Bangladesh (0.42, 95% UI: 0.33–0.52) experienced the most substantial relative increases in ASPR (S1 and S2 Tables). The national levels of ASIR were detailed in S1 and S2 Tables. Significant upward trends from 1990 to 2021 were observed in Slovakia (0.40, 95% UI: 0.33–0.48), Serbia (0.35, 95% UI: 0.29–0.42), and Bulgaria (0.31, 95% UI: 0.25–0.38). Conversely, Senegal, Burkina Faso, and Liberia demonstrated notable reductions at −0.40, −0.37, and −0.34 per 100,000 populations, respectively.

The ASMR for the condition ranged from 0.23 to 0.32 per 100,000 population. In 2021, Mongolia (1.59, 95% UI: 0.91–2.05), Georgia (1.01, 95% UI: 0.79–1.27), and Tajikistan (0.81, 95% UI: 0.53–1.09) had the highest ASMR. Over the study period, the countries with the largest increases in ASMR were Latvia (3.52, 95% UI: 2.65–4.42), Republic of Moldova (2.54, 95% UI: 1.93–3.39), Georgia (1.72, 95% UI: 0.94–2.81), and Mauritius (1.71, 95% UI: 1.39–2.06). The most significant decreases were seen in Puerto Rico (−0.83, 95% UI: −0.86 to −0.79), Guatemala (−0.78, 95% UI: −0.82 to −0.72), Costa Rica (−0.76, 95% UI: −0.79 to −0.72), and Greenland (−0.75, 95% UI: −0.84 to −0.38) (S1 and S2 Tables). Detailed data on DALYs were provided in S1 and S2 Tables. Additionally, the correlation analyses of SDI and 204 countries were presented in S3 Fig.

### 3.4. Age and sex patterns

Age-stratified analyses of PAH burden in 2021 revealed distinct non-linear trends. The ASPR showed a progressive age-dependent escalation, peaking in the 75–79 years group, with consistently higher rate observed among females across all age strata. A similar age-related increase was noted for ASIR. Interestingly, males exhibited higher ASMR than females during infancy and between 55–94 years of age, while no significant gender difference were observed between ages 2 and 54. This study also showed that ASDR had the highest levels in infancy and a lower trend in ages 2–54, while increasing with age after 40 years (S4 Fig and S3 Table).

**Fig 1:**
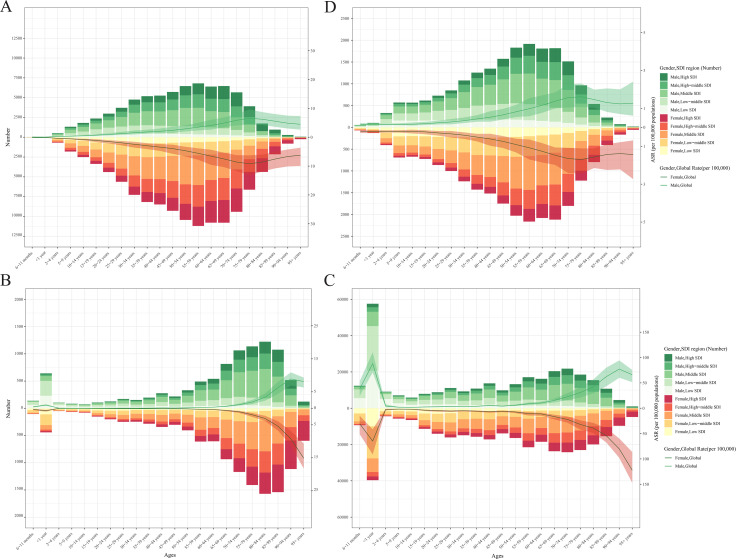
The age-specific number and rate of PAH burden by SDI regions in 2021. **(A)** Prevalence; **(B)** Incidence; **(C)** Mortality; **(D)** DALYs.

Notably, ASPR and ASIR were significantly elevated in middle-aged and older populations compared to younger groups across all SDI regions. An ascending trend in ASMR and ASDR was also observed in infant populations over the study period. The distribution of PAH cases shifted toward older age groups in higher SDI regions. Furthermore, the DALYs exhibited a consistent age-dependent increase across all SDI regions (Fig 1).

### 3.5. Temporal joinpoint analysis

**Fig 2:**
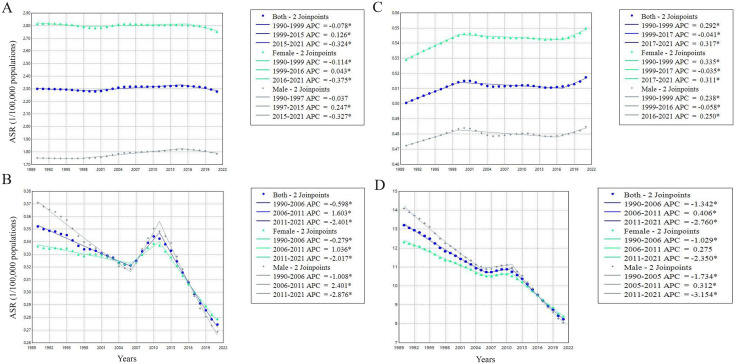
Joinpoint regression analysis of PAH burden temporal trends, 1990-2021. **(A)** Age-standardized prevalence rates; **(B)** Age-standardized incidence rates; **(C)** Age-standardized mortality rates; **(D)** Age-standardized DALYs rates. APC indicates year-to-year variations, while AAPC gives the average trend over specified periods. * showed *P* <0.05. Abbreviations: APC, annual percent change; AAPC, average annual percent change; CI, confidence interval.

Joinpoint regression analysis indicated that the global ASPR of PAH remained stable from 1990 to 2021 (AAPC = −0.02%, 95% CI: −0.06% to 0.02%; *P* = 0.260), though a significant downward trend was observed between 2015–2021 (APC = −0.32%, 95% CI: −0.46% to −0.19%; *P* < 0.001). In contrast, the ASIR exhibited a significant upward trend over the entire period (AAPC = 0.10%, 95% CI: 0.08% to 0.12%; *P* < 0.001), with the most pronounced increase occurring from 2017 to 2021 (APC = 0.32%, 95% CI: 0.19% to 0.45%; *P* < 0.001). The ASMR showed a overall decline from 1990 to 2021 (AAPC = −0.83%, 95% CI: −0.90% to −0.77%; *P* < 0.001), despite a marked increase during 2006−2011 (APC = 1.60%, 95% CI: 1.25% to −1.96%; *P* < 0.001). Similarly, the ASDR decreased over all during the study period (AAPC = −1.52%, 95% CI: −1.59% to −1.46%; *P* < 0.001), but an upward trend was reported between 2006 and 2011 (APC = 0.41%, 95% CI: 0.07% to 0.75%; *P* = 0.02) (Fig 2 and S4 Table).

### 3.6. Decomposition analysis

**Fig 3:**
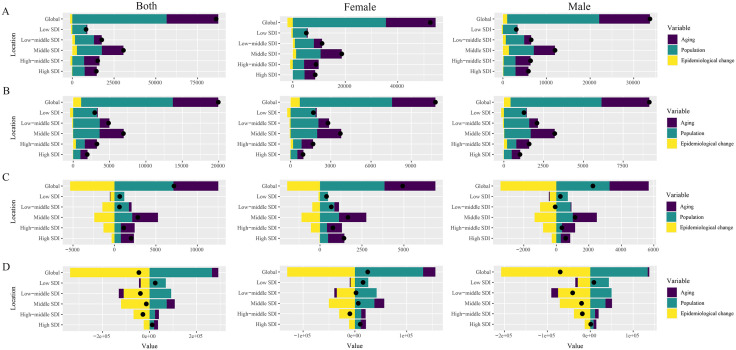
Decomposition analysis of relative contribution according to aging, population growth, and epidemiological change from 1990 to 2021 at the global level by SDI quintile and sexes subgroups. **(A)** Prevalence; **(B)** Incidence; **(C)** Mortality; **(D)** DALYs. Shading denotes represented overall difference, indicating the gap of the population between 2021 and 1990. Columns of different colors represent the contribution of decomposition factors to the overall difference.

Decomposition analysis contributors to PAH burden revealed distinct patterns across SDI quintiles. Population aging was the predominant driver in high-middle SDI regions (60.72%), whereas population growth had the greatest influence in low SDI regions (103.11%). Epidemiological change accounting for 9.07% for prevalence within middle SDI regions. A similar decomposition of incidence metrics showed consistent trends, alongside significant gender disparities, with a higher disease burden observed among females. DALYs were substantially influenced by epidemiological change, particularly in middle SDI regions, which manifested an extraordinary 878.94% escalation. In contrast, mortality analysis demonstrated a negative contribution from epidemiological change in low-middle SDI regions (−241.97%) (Fig 3 and S5 Table).

### 3.7. Cross-country inequality analysis

**Fig 4:**
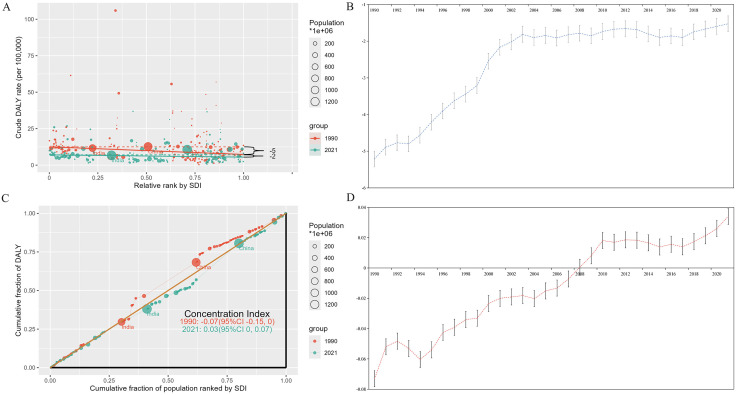
SDI-related health inequality for DALYs of PAH worldwide, 1990 and 2021. **(A)** A Scatter plot of age-standardized DALYs rates and Slope index of inequality in 1990 and 2021; **(B)** Change of slope index of inequality from 1990 to 2021; **(C)** Lorenz curve and concentration index in 1990 and 2021; **(D)** Change of concentration index from 1990 to 2021. CI: confidence interval.

Cross-country inequality analyses showed that both absolute and relative SDI-related inequalities in PAH burden widened over time (Fig 4 and S6 Table). Notably, DALYs disproportionately concentrated in countries with higher socio-demographic development levels. The slope index of inequality further quantified this disparity, showing the gap in DALYs per 100,000 populations between the highest SDI and the lowest SDI countries increased from −5.21 (95% CI: −7.50 to −2.93) in 1990 to −1.52 (95% CI: −3.03 to −0.02) in 2021. Additionally, the concentration index shifted from −0.07 (95% CI: −0.15 to <0.001) in 1990 to 0.03 (95% CI: −0.004 to 0.07) in 2021.

### 3.8. Predictive analysis on global burden of PAH to 2050

**Fig 5:**
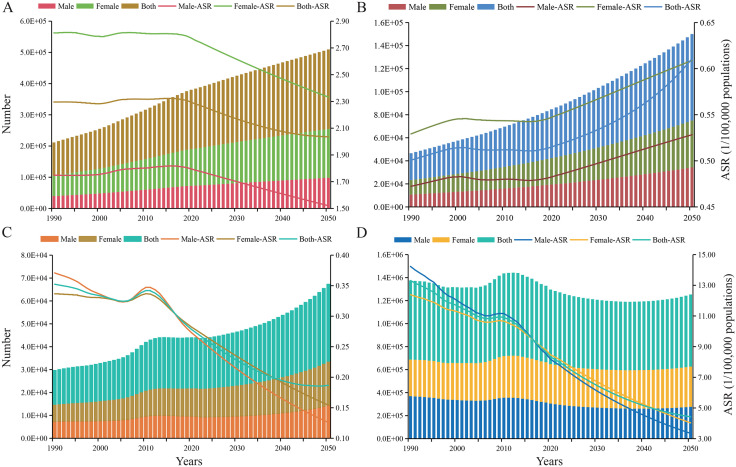
The predicated number and age-standardized rates of prevalence (A), incidence (B), mortality (C), and DALYs (D) to 2050. ASR: age-standardized rates.

Prediction indicted a slight decline in ASPR from 2.28 per 100,000 populations in 2021 to approximately 2.04 per 100,000 populations by 2050. The ASIR was expected to remain relatively stable. The ASMR and ASDR were predicted to decrease from 0.27 to 0.19 per 100,000 populations and from 8.15 to 4.42 per 100,000 populations over the same period, respectively. Despite these declining rates, the absolute numbers of PAH cases were predicted to continue rising (Fig 5 and S7 Table). Although the gender-based gap appeared to narrow over time, females consistently showed higher rates compared to males.

## 4. Discussion

PAH remains a formidable global health challenge. This study provided a comprehensive assessment of global, regional, and national PAH burden from 1990 to 2021, offering the foundation for medical resource allocation and future research in prevention and treatment.

Although the incident cases of PAH increased over the study period, reaching 1,036,494 (95% UI: 840,231−1,246,849) globally in 2021, the ASMR and ASDR was observed to decrease, which likely reflected improvements in early screening, diagnosis, and treatment, particularly PAH associated with congenital heart disease (CHD). Notably, the highest burden for ASIR, ASMR, and ASDR were concentrated in low to middle SDI regions, underscoring pronounced socioeconomic disparities. The insidious nature of PAH often leaded to delayed diagnosis, especially in regions with limited healthcare access. In low to middle SDI regions, factors such as uncontrolled infections, barriers to specialized care converge to exacerbate disease burden [18–20]. For instance, limited availability of RHC and advanced therapeutic options such as targeted medications, interventional procedures, and cardiothoracic surgery, likely contributed to higher prevalence and incidence in Latin America and Sub-Saharan Africa [21]. Moreover, etiological characteristics contributed to the geographic variation in the burden of PAH. CHD and human immunodeficiency virus were dominant causes in Africa, whereas schistosomiasis was one of key contributors in South America [22]. In contrast, high SDI regions benefited from robust healthcare infrastructures, effective public health policies, and greater disease awareness, which facilitated early detection and management, resulting in lower mortality and DALYs [6,23]. For instance, the lower burden was observed in high SDI regions such as High-income Asia Pacific and Western Europe, likely reflecting the importance and necessity of primary and secondary prevention [24].

Despite the decline in age-standardized rates, the absolute number of cases remained substantially high, largely due to population aging and growth, as well as the global transition from communicable to non-communicable diseases [25]. These demographic transitions strained healthcare resources and compounded existing disparities. Additionally, environmental factors such as chronic exposure to air pollution and the rising prevalence of cardiovascular comorbidities further amplified the disease burden [1,26].

Consistent with the pathophysiology of PAH, disease burden increased with age, peaking in older adult groups, likely due to cumulative risk factor exposure and age-related pulmonary vascular changes such as chronic obstructive pulmonary disease [27]. Interestingly, the highest ASDR was observed among infants, reflecting the impact of severe congenital cardiovascular anomalies such as tetralogy of Fallot and Fontan circulation [28,29]. A consistently elevated burden of PAH was also observed among females, especially older adults, which may be linked to sex-specific hormonal pathways in different pathophysiological mechanisms [30]. Morris et al. [31] found female sex acted as an independent risk factor for PAH and highlighted the interactions between estrogens and BMPR2 mutation. Furthermore, dysfunctional estrogen metabolism, resulting in increase of 16OHEs levels, has been implicated in the pathogenesis of PAH [31].

This study performed the cross-national comparisons and longitudinal analysis of burden and trends of PAH based on the comprehensive and standardized GBD 2021 data. However, several limitations should be considered in this study. First, the accuracy of GBD estimates relied on the availability and quality of underlying data, which remained uneven across regions, particularly in regions with underdeveloped health information systems. Second, although the GBD employed rigorous statistical methods such as hierarchical Bayesian models to mitigate data gaps, there remained subject to uncertainty. Finally, constrained accessibility to healthcare resources limited the diagnosis of PAH in lower SDI regions, potentially leading to an underestimation of disease burden.

In conclusion, while age-standardized rates of PAH have declined globally, the absolute burden remained high, with disproportionate impacts on females, older populations, and low SDI regions. Addressing these disparities required coordinated efforts among healthcare providers, policymakers, and researchers to enhance diagnostic capacity, develop prevention strategies, and target treatment strategies in different genetic, environmental, and clinical contexts.

## Supporting information

S1 Table ASPR, ASIR, ASMR and age-standardised DALYs rates of PAH for both sex by country, 2021. (XLSX)

S2 Table Age-standardised prevalence, incidence, mortality, and DALYS changes rates of PAH for both sex by country, 1990–2021. (XLSX)

S3 Table Global age structure of PAH prevalence, incidence, mortality, and DALYs both males and females, 2021. (XLSX)

S4 Table Temporal joinpoint analysis ASPR, ASIR, ASMR, and age-standardized DALYs rates for PAH, 1990–2021. (XLSX)

S5 Table Frontier analysis of mortality and age-standardised DALYs rates for PAH in 2021. (XLSX)

S6 Table Decomposition analysis in prevalence, incidence, mortality, and DALYs of PAH from 1990 to 2021. (XLSX)

S7 Table Inequality for the DALYs of PAH worldwide from 1990 to 2021. (XLSX)

S8 Table The predicated global case number and rate of prevalence, incidence, mortality, and DALYs by 2050. (XLSX)

S1 Fig Trends in PAH prevalence, incidence, mortality, and DALYs among 32 years. (DOCX)

S2 Fig The associations between SDI and PAH burden across global and 21 GBD regions. (DOCX)

S3 Fig The associations between SDI and PAH burden across 204 GBD countries and territories. (DOCX)

S4 Fig Age-standardized rate analyses for PAH of global by sex and age group in 1990 and 2021. (DOCX)

S5 Fig Frontier analysis based on SDI and ASMR. (DOCX)
