# Supplementary material for: Global, regional, and national burden of pulmonary arterial hypertension from 1990 to 2021 and projection to 2050: A systematic analysis for the global burden of disease study 2021
Source: PLoS One. 2025 Dec 29;20(12):e0338335. doi: 10.1371/journal.pone.0338335 (PMC12747407; doi:10.1371/journal.pone.0338335)
Supplement: S1 Fig — (DOCX) [file pone.0338335.s009.docx]

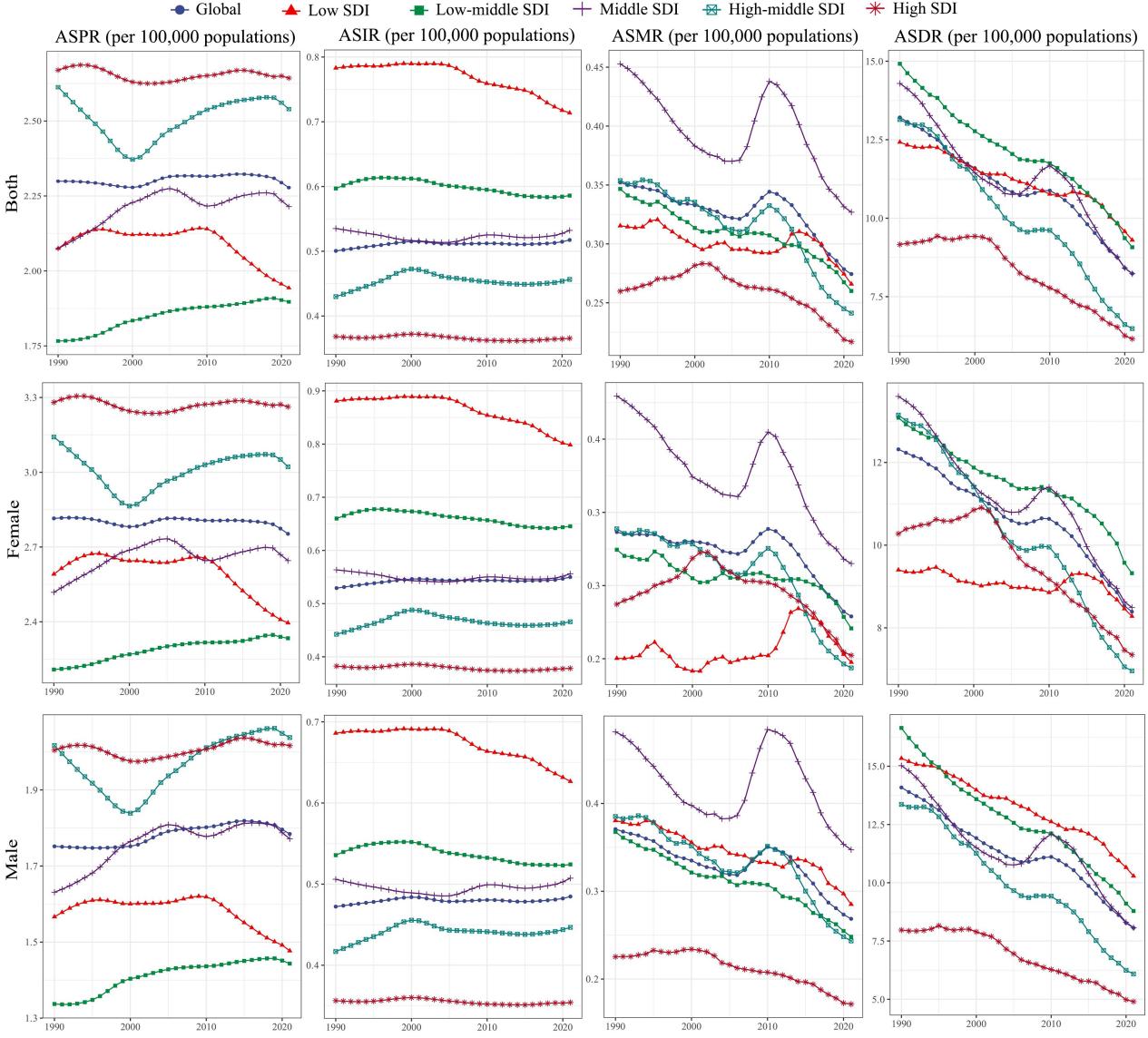


**S1 Fig. Trends in PAH prevalence, incidence, mortality, and DALYs among 32 years.** Abbreviations: ASPR, age-standardized prevalence rates; ASIR, age-standardized incidence rates; ASMR, age-standardized mortality rates; ASDR, age-standardized DALYs rates; DALYs, disability-adjusted life years.
