# Supplementary material for: Global, regional, and national burden of pulmonary arterial hypertension from 1990 to 2021 and projection to 2050: A systematic analysis for the global burden of disease study 2021
Source: PLoS One. 2025 Dec 29;20(12):e0338335. doi: 10.1371/journal.pone.0338335 (PMC12747407; doi:10.1371/journal.pone.0338335)
Supplement: S3 Fig — (DOCX) [file pone.0338335.s011.docx]

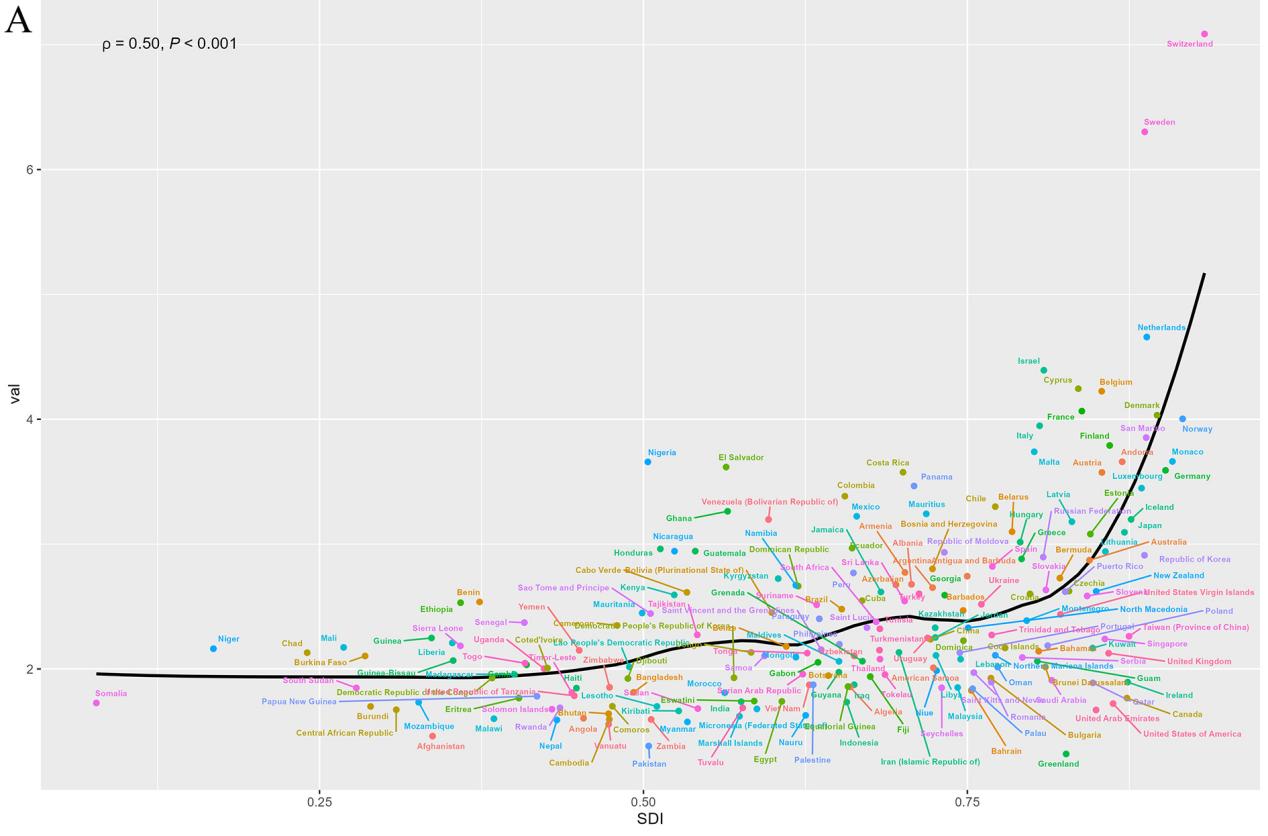


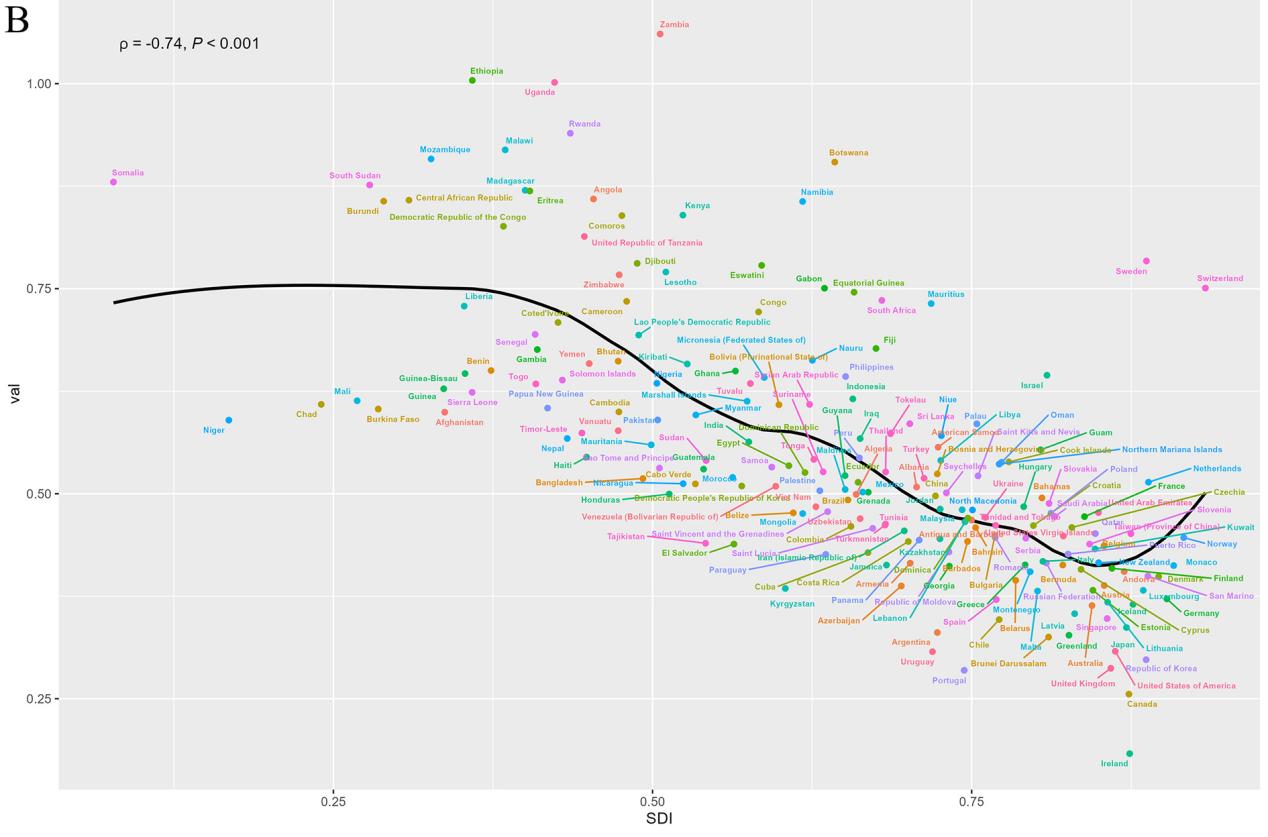


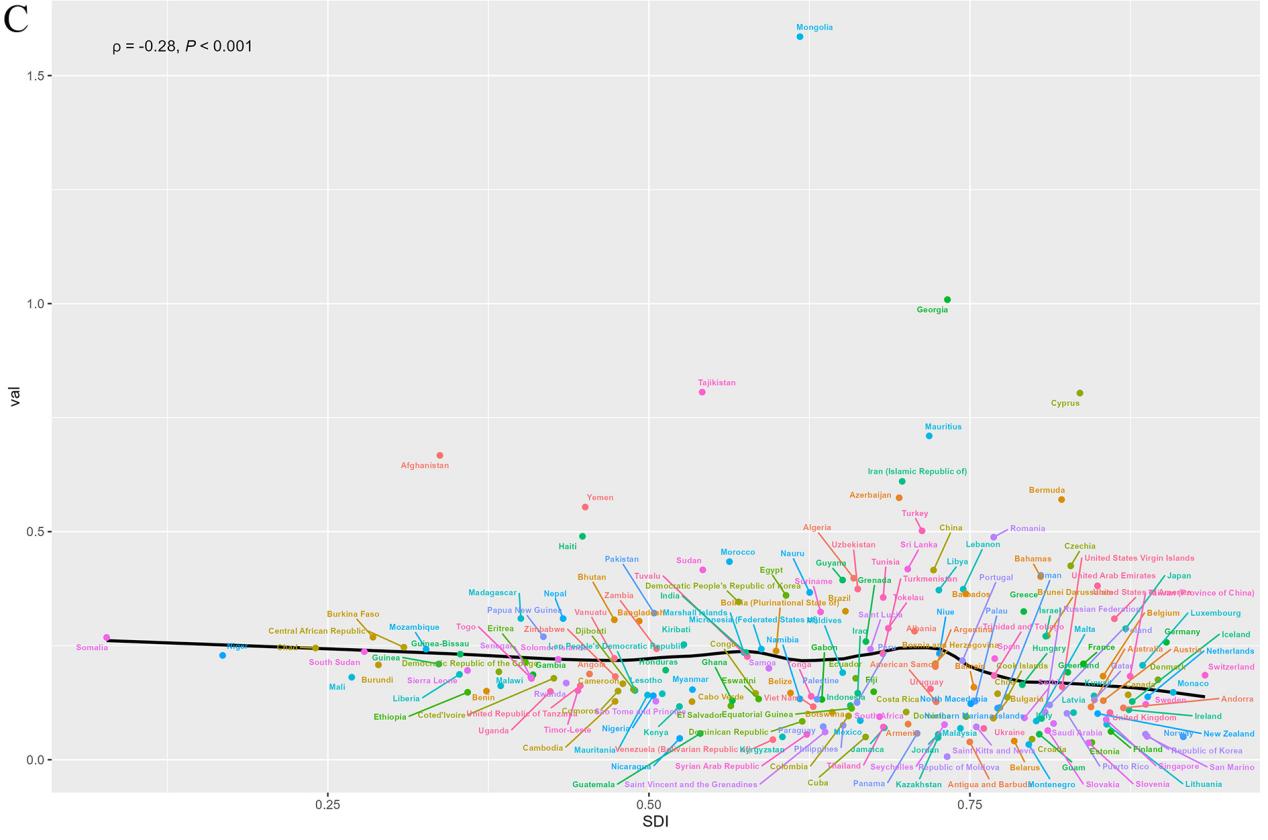


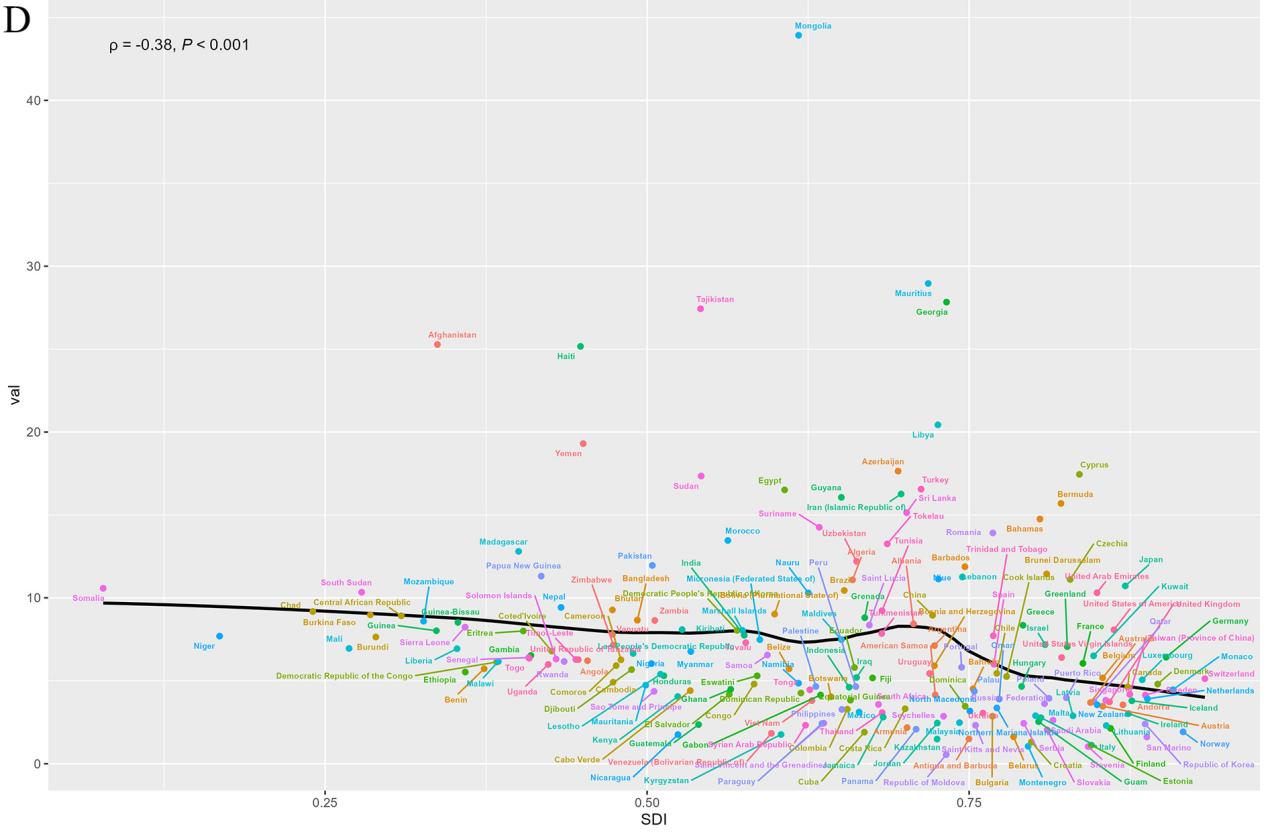


**S3 Fig.** **The associations between SDI and PAH burden across 204 GBD countries and territories.** (A) Age-standardized prevalence rates; (B) Age-standardized incidence rates; (C) Age-standardized mortality rates; (D) Age-standardized DALYs rates.
