# Supplementary material for: Global, regional, and national burden of pulmonary arterial hypertension from 1990 to 2021 and projection to 2050: A systematic analysis for the global burden of disease study 2021
Source: PLoS One. 2025 Dec 29;20(12):e0338335. doi: 10.1371/journal.pone.0338335 (PMC12747407; doi:10.1371/journal.pone.0338335)
Supplement: S4 Fig — (DOCX) [file pone.0338335.s012.docx]

**
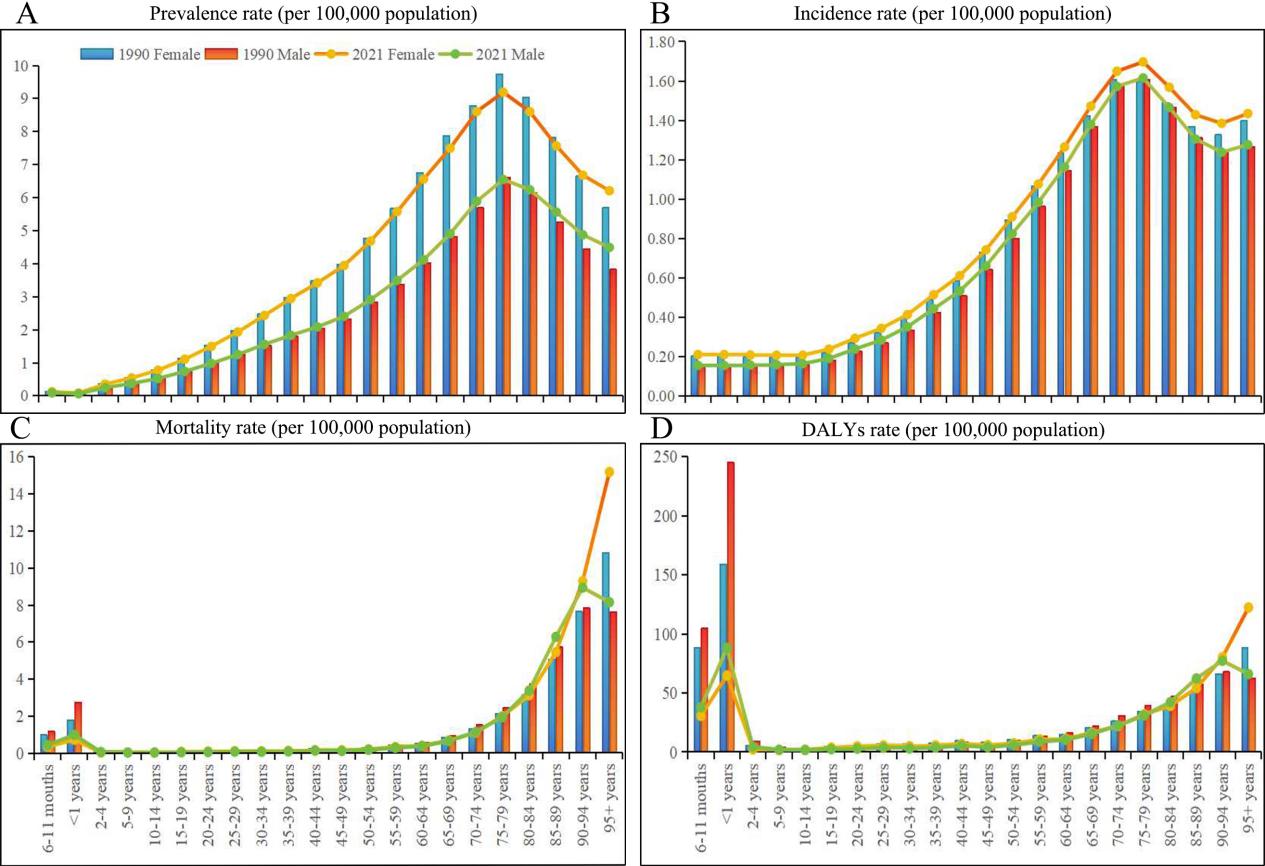
**

**S4 Fig. Age-standardized rate analyses for PAH of global by sex and age group in 1990 and 2021.** (A) Prevalence; (B) Incidence; (C) Mortality; (D) DALYs.
