# Supplementary material for: Global, regional, and national burden of pulmonary arterial hypertension from 1990 to 2021 and projection to 2050: A systematic analysis for the global burden of disease study 2021
Source: PLoS One. 2025 Dec 29;20(12):e0338335. doi: 10.1371/journal.pone.0338335 (PMC12747407; doi:10.1371/journal.pone.0338335)
Supplement: S5 Fig — (DOCX) [file pone.0338335.s013.docx]

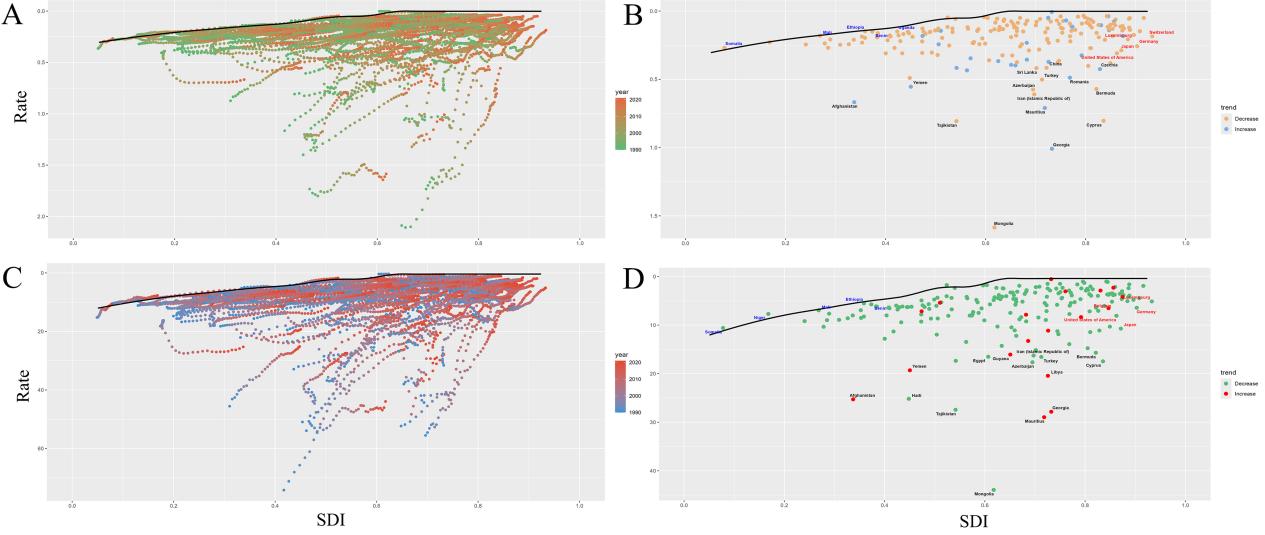


**S5 Fig.** (A) Frontier analysis based on SDI and ASMR from 1990 to 2021; (B) Frontier analysis based on SDI and ASMR in 2021. (A, C) Frontier analysis based on SDI and ASMR and ASDR from 1990 to 2021. Color scale represents the years from 1990 depicted in blue and to 2021 depicted in red. And the frontier is delineated in solid black color. (B) Frontier analysis based on SDI and age-standardized PAH DALYs rate in 2021. The frontier is delineated in solid black color; countries and territories are represented as dots. The top 15 countries with the largest effective difference (largest PAH DALYs gap from the frontier) are labeled in black; examples of frontier countries with low SDI (<0.5) and low effective difference are labeled in blue; examples of countries and territories with high SDI (>0.85) and relatively high effective difference for their level of development are labeled in red. Green dots indicate an increase in ASDR from 1990 to 2021; red dots indicate a decrease in ASDR between 1990 and 2021.
